# Probit analysis of comparative assays on toxicities of lead chloride and lead acetate to *in vitro* cultured human umbilical cord blood lymphocytes

**DOI:** 10.1515/intox-2015-0007

**Published:** 2015-03

**Authors:** Rajashree Patnaik, Rabindra N. Padhy

**Affiliations:** Central Research Laboratory, IMS & Sum Hospital, Siksha ‘O’ Anusandhan University, Kalinga Nagar, Bhubaneswar, Odisha, India

**Keywords:** cytotoxicity, genotoxicity, lead chloride, lead acetate, AO/EB staining, MTT assay, comet assay

## Abstract

This work describes that cytotoxicity of lead chloride and lead acetate to *in vitro* cultured lymphocytes from human umbilical cord blood, using four monitoring methods namely, trypan blue staining, acridine orange/ethidium bromide staining, 3-[4,5-dimethylthiazol-2-yl] 2,5-diphenyl tetrazolium bromide (MTT) and neutral red uptake assays; lead genotoxicity to lymphocytes was monitored by comet assay. The MIC value in each method was invariably 300 mg/L for PbCl_2_. Lethal concentration_25_ (LC_25_) values were almost in an agreeable range: 691.83 to 831.76 mg/L; LC_50_ values in each method were almost in the range: 1174.9 to 1348.9 mg/L; LC_100_ values were in the range: 3000 to 3300 mg/L, for lead chloride. Similarly, The MIC value in each method were invariably 150 mg/L; LC_25_ values were almost in the range: 295.12 to 371.53 mg/L; LC_50_ values were in the range: 501.18 to 588.84 mg/L; LC_100_ value was 1500 mg/L in all assays, for lead acetate. The comet assay also indicated that the LC_100_ values were 3300 mg/L lead chloride and 1500 mg/L lead acetate. Thus, both cytotoxicity and genotoxicity were recorded at 3300 mg/L lead chloride and 1500 mg/L lead acetate with lymphocytes.

## Introduction

Without any role in metabolism, lead or *Plumbum* (Pb, Group 2b) is a toxic heavy metal. Effluents from industries dealing with paper, food canning, battery and cosmetics as well as mining industry, oil refineries and coal mine establishments mainly add Pb compounds to the total environment. Of all Pb compounds produced in the United States, for example, a 4% (80,000 tons/year) approximately is made into bullets; consequently, 58,300 tons/year Pb approximately from shot and munitions get deposited onto landscape through shooting activities (USEPA, 2001). Pb is present in environments in soluble, PbCl_2_, CH_3_COOPb (lead acetate) and PbCO_3_, as well as insoluble PbS, Pb_3_(PO_4_)_2_, PbO and PbSO_4_ forms, apart from spillage as ore powders from establishments dealing with Pb-minerals, hydrocerussite, cerussite and massicot. Moreover, leaching of water soluble Pb compounds in soil in the rainy season causes the Pb-sorption by biota (Sharma *et al*., 2010, Moussa *et al*., 2008, Campbell *et al*., 2004).

From studies on lead toxicity with Wistar rats by injecting lead acetate solution at 15 mg/kg body weight, a significant decrease in the level of superoxide dismutase activity and increase in plasma bilirubin level were recorded (Berrahal *et al*., 2007, Rogers *et al*., 2003). A report in rat system showed that the ingestion of Pb induced stimulation in glutamic-pyruvic transaminase and glutamic-oxalacetic transaminase activity; the cholinesterase activity was inhibited, while hyperglycemia was induced in lead acetate toxicity; in blood, metallic Pb reduced hemoglobin contents and red blood cell (RBC) count as well as the plasma levels of T_3_, T_4_ and white blood cell count had decreased (Ewis *et al*., 2012). In Wistar rats the relative retention of lead acetate by the issues was determined as follows: lungs>spleen>stomach>kidney> blood>heart in males and in females as spleen>stomach> heart>kidney>blood>lungs in the ip-route, and those were kidney>lungs>stomach>blood>heart>spleen, in males and kidney>lungs>stomach>blood>heart>spleen in females in the oral route (Jarrar & Taib, 2012). A low level exposure to lead acetate reported to cause adverse effects on neurons of zebrafish embryos (Zhang *et al*., 2012).

A variety of toxic effects caused by Pb compounds had been identified during gestation and lactation in animals and humans (Bunn *et al*., 2001). Lead exposure was demonstrated to occur both through respiratory and gastro-intestinal tracts (Park *et al*., 2008). About a 60% of ingested lead was reported to be absorbed by empty stomach, but along with food a lesser amount (15-20%) was absorbed. Obviously, blood is the inadvertent route of the metal mobility to brain, liver, bone marrow and testis. Furthermore, child-neuro development and adult brain cells were recorded to be affected by lead toxicity (Bellinger, 2008). It was reported that because of the exposure of the mother to pollutants, the fetus in womb was affected by lead poisoning with the eventual impairment of the natural development of immune system in the postnatal life (Bishoyi & Sengupta, 2006). Ultimately, Pb gets stored in bone inducing decrease in bone mineral density (Lee *et al*., 2010). Monitoring of levels of renal thioredoxin reductase-1 activity in rats exposed to 25 mg/kg lead acetate was recorded to induce renal damage (Conterato *et al*., 2014). And it was demonstrated that lead acetate at 1 and 1.5 g/L caused male sterility in rats in a dose dependent manner (Wang *et al*., 2013). Increase of low density lipoprotein cholesterol, atherogenetic index and coronary heart disease index levels with exposure to lead acetate in broiler chicken were, 45.24%, 100%, 16.66% compared to the control group, in a study (Karimi *et al*., 2013).

Apart from gastro-intestinal disturbances in humans, lead toxicity causes restlessness, irritability, headache, hepatic and renal damage, hypertension, hallucination and encephalopathy; in blood basophilic stippling and decreased hemoglobin synthesis occur due to Pb (Anonymous, 2007). Age related impairments in behavioral functions had been linked to lead poisoning that caused changes in neurotransmitter levels in brain tissue (Chand *et al*., 2014). Lead induced biochemical and structural changes occur in liver causing cataclysmic alterations by oxidative stress, apoptosis and mitogen activated protein kinase (Mujaibel & Kilarkaje, 2013, Samaraghandian *et al*., 2013). Particularly, oxidative stress had been reported to be one of several important possible mechanisms in lead toxicity (Shalan *et al*., 2005) with the generation of hydroxyl radical, hydrogen peroxide, superoxide anion and lipid peroxidase, *etc*. (Soltaninejad *et al*., 2003).

A single report on the use of human umbilical cord mesenchymal stem cells had been recorded monitoring lead trioxide toxicity at <10 mM; lead acetate too had toxic effect on the self-renewal, multipotent differentiation potential and hematopoiesis-promoting function of mesenchymal stem cells from umbilical cord blood (UCB) (Sun *et al*., 2012). Pb had been reported to have toxicity to hematopoietic system by interfering with hemoglobin synthesis and erythrocyte morphology. In this study, cell count, 3-[4,5-dimethylthiazol-2-yl] 2,5-diphenyl tetrazolium bromide (MTT) assay, apoptosis assay, osteogenic differentiations were recorded to be affected by lead trioxide. Alkaline phosphatase enzyme was lower than control group (Sun *et al*., 2012). DNA damage by lead acetate exposure was reported in the rabbit model at 15 mg/kg; infraction of the kidneys and intranuclear specific inclusion bodies in liver and kidney were detected (Ahmed *et al*., 2012).

This work describes toxicity of PbCl_2_ and lead acetate to *in vitro* cultured lymphocytes from human UCB. Cyto-toxicity was monitored using trypan blue (TB) and acridine orange/ethidium bromide (AO/EB) staining, MTT and neutral red uptake (NRU) assays; genotoxicity was monitored by comet assay. Cord blood being a waste blood, its use in experiment in ‘predictive toxicity’, such as herein, should not warrant ethical issues. Predictive toxicology is an offshoot of pharmacology describing concepts related to toxic effects of newer chemicals and prospective drugs in model systems to predict health-risk assessments, before institutional recommendation as a drug and other uses. The underlying method could well be adopted for environmental toxicants, for planning well-being of human health. Differentiated cells obtained from a mass of lymphocytes could be able to mimic some actions related to chemical kinetics operative naturally in metabolism as a response to the chemical. Thus, this would help examine the probable role of some toxic chemicals in human system. Lymphocytes help the defense mechanism of the body against infectious agents, due to their ability to distinguish the body’s own cells from the foreign ones.

## Materials and methods

### Collection of lymphocytes

**Figure 1:**
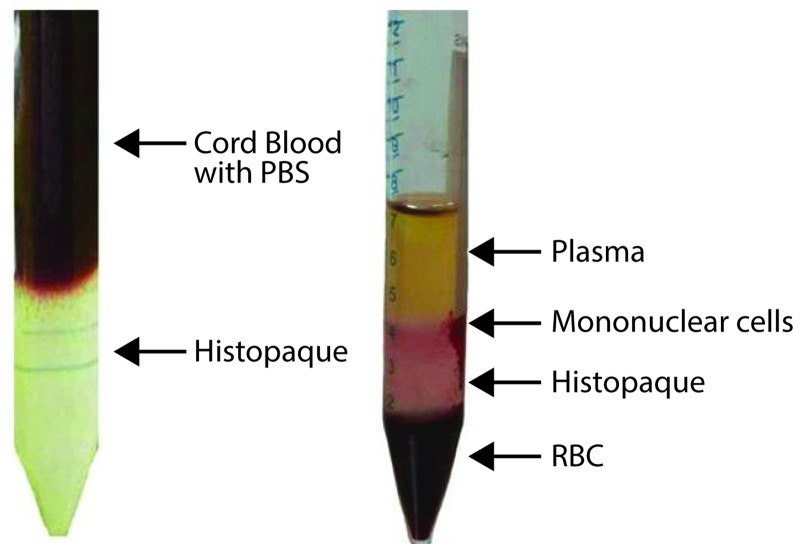
Density gradient centrifugation of human umbilical cord blood.

With an aliquot of 100 or 250 μL 1,000 IU heparin (HiMedia, Mumbai), in a sterile 15 or 50 mL size falcon tube (Tarson, Kolkata), the UCB sample was collected according to volume, immediately after the delivery of an infant and was stored at 4 ^°^C till use. Lymphocytes were isolated immediately or within at the best 24 hrs after the collection of UCB. For the isolation of lymphocytes, the collected UCB sample was diluted with phosphate buffered saline (PBS) solution in 1:1 proportion. The mixture was loaded carefully into a centrifuge tube for over-layering on Histopaque (Sigma, Mumbai) for the separation of lymphocytes, which was one-third the total volume of the mixture. The mixture was centrifuged at 1,800 rpm for 25 min at 22–24 °C; and four heavy to light layers, RBC, Histopaque, buffy coat and plasma were seen (Figure 1). Mononuclear cells in the buffy coat layer were taken out carefully from the tube. To the separated cells, another aliquot of PBS in the 1:1 ratio was added, mixed gently and re-centrifuged at 2,000 rpm for 5 min. The pellet of lymphocytes was taken for culturing and subsequently cell counts were done using a haemocytometer.

### Growth of lymphocytes

**Figure 2:**
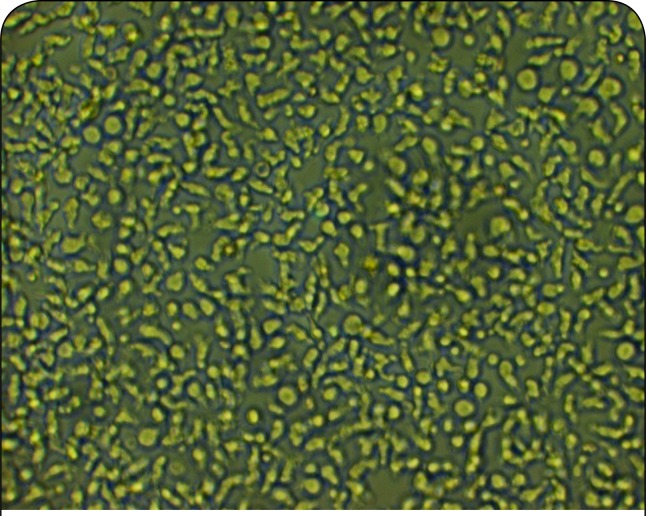
Photomicrograph of growing lymphocytes; magnification, ×400.

After separation, UCB-derived lymphocytes were diluted to the density of 1×10^6^ cells/mL with a required volume of Dulbecco’s modified Eagle’s medium (DME medium-lowglucose, HiMedia, Mumbai), and were loaded into a 6-well culture plate (Tarson); DME medium contained 15% fetal bovine serum (Sigma), 1% penicillin-streptomycin and 1% sodium pyruvate, along with graded concentrations of PbCl_2_ or lead acetate solution for growth. The stock solution was prepared by dissolving 1000 mg of PbCl_2_ or lead acetate (Sigma) in aliquots of 100 mL of triple distilled water for the final concentration of 10,000 mg/L, and the stock solution was stored at 4^º^C, for further use. The volume of 2 mL in total mixture was maintained in each well of the culture plate; the cells were incubated with different concentrations of PbCl_2_ (0, 300, 600, 900, 1200, 1500, 1800, 2100, 2400, 2700, 3000 and 3300 mg/L), or lead acetate (0, 150, 300, 450, 600, 750, 900, 1050, 1200, 1350 and 1500 mg/L) in an incubator at 37 °C in 5% atmospheric CO_2_ concentration for 24 hrs for growth (Figure 2).

### Assessment of cyto- and genotoxicity

The viability of lymphocytes grown in the presence of PbCl_2_ and lead acetate was assessed using two staining procedures, with TB and AO/EB, using a phase-contrast (Magnus, New Delhi) and fluorescent microscope (Magnus), respectively. MTT assay and NRU assay were also carried out as the standard procedures for monitoring the live cell density. Comet assay was done for assessing Pb-induced nuclear damages.

### TB staining

TB solution was prepared in PBS at the concentration of 0.4 g/mL. For the study of cell viability to the *in vitro* grown mass of lymphocytes, TB solution was added at the 1:1 ratio; the mixture was kept in an incubator for 2 min at 37 °C and cells were observed under the phase-contrast microscope at the 400× magnification. The live cells remained unstained, whereas the nuclei of the dead cells were blue in appearance, as TB enters dead cells and stains the nuclei to blue, as it is a membrane permeable dye.

### AO/EB staining

**Figure 3:**
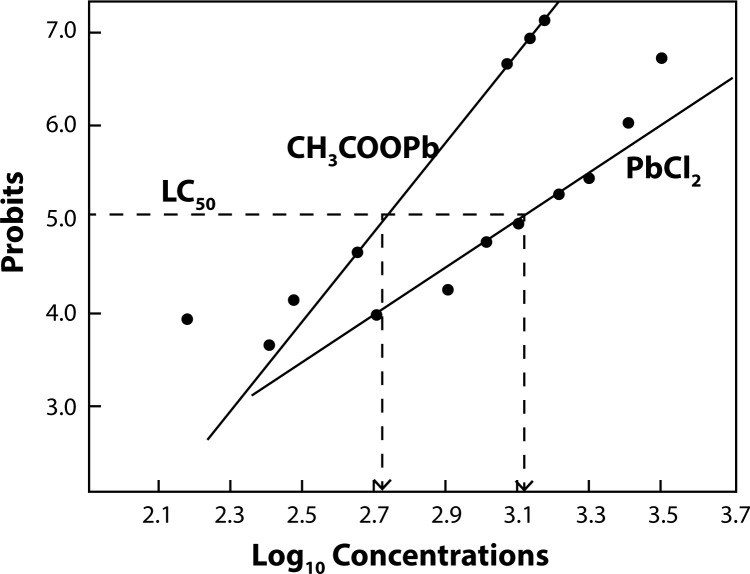
Probits of percentage lethality values plotted against log_10_ concentrations of PbCl_2_ and CH_3_COOPb (lead acetate) in the toxicity study of lymphocytes by MTT assay; each line is fitted by eye; three pairs of log_10_ concentration values were determined taking probit points, 4.3255 (LC_25_), 5.0000 (LC_50_) and 5.6745 (LC_75_).

**Figure 4:**
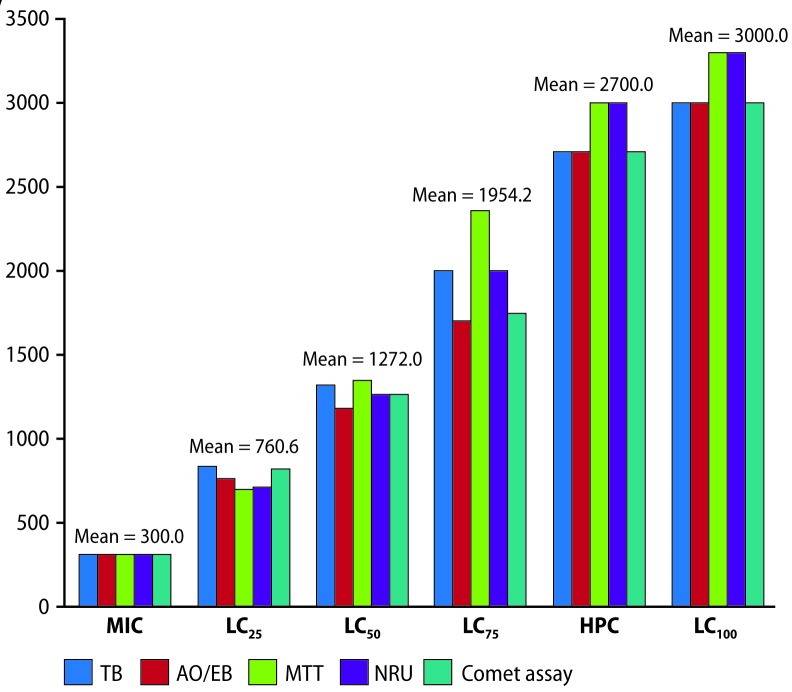
Histogram of MIC, LC_25_, LC_50_, LC_75_, HPC and LC_100_ values corresponding to TB, AO/EB, MTT, NRU and comet assay methods (see text for abbreviations) after the lymphocytes treated with PbCl_2_. M= mean value (mg/L).

The AO/EB solution was prepared in PBS at the concentration of 100 μg/mL and is applied to cultured lymphocytes after a 24 hrs of incubation in presence of graded concentrations of any of the two Pb compounds. When observed under the fluorescent microscope at 400×, green colour indicated live cells, whereas cells with orange and red colour were apoptotic and necrotic cells, respectively (Figure 3). AO is taken by both live and dead cells and emits green fluorescence. EB is only taken up by cells when the integrity cytoplasmic membrane is lost and stains nucleus orange. Hence live cells, apoptotic cells and necrotic cells were green, orange and red in appearance respectively. Toxicity values were obtained with different concentrations of PbCl_2_ and lead acetate (Figure 4). Percent lethality (PL) values of the third repeated experiment were transformed to probit values by Finney’s method, which were potted against corresponding log_10_ values of PbCl_2_ and lead acetate concentrations, as exemplified before (Rath *et al*., 2011). Probits of observed lethality percentage values were from statistical tables of probit transformation.

### MTT assay

The MTT stock solution was prepared at the concentration of 5 mg/mL in PBS. After 24 hrs of toxin treatment in 6-well culture plate, 80 μL of MTT stock solution was added to each well to study the toxicity effect. The plate was kept in an incubator (37 °C, 5% CO_2_) for 4 hrs and it was found that media containing cells and toxin got converted to blue colour after incubation with MTT. The mixture was gently centrifuged at 1,000 rpm for 10 min at 22 °C. The supernatant was removed and the pellet was dissolved in 1 mL aliquot of 100% dimethyl sulfoxide (DMSO) and the suspension was kept in an incubator for 1 hr. Optical density_570_ of the suspension with the purple colour was measured with a spectrophotometer. Percentage of cell density was calculated as follows:

$$100\times ({\text{OD}}_{\text{sample}}-{\text{OD}}_{\text{blank}})/{\text{OC}}_{\text{control}}$$

MTT in DMSO solution was taken as the blank. Probits of observed lethality percentage values and log_10_ concentration values of chemical were used for analysis of toxicity.

### NRU assay

Neutral red solution was prepared in serum-free DME medium at the concentration of 100 μg/mL. After 24 hrs growth of lymphocytes in the presence of a toxin in a 6-well culture plate, an aliquot of 200 μL of neutral red dye solution was added to each well. The plate was kept in an incubator (37 °C, 5% CO_2_) for 3 hrs. Indeed, lysosomes of viable cells absorb the dye and the dead cells remain unstained. Thereafter, an aliquot of 1 mL of separately prepared neutral red desorption solution (1% acetic acid, 50% ethanol and 49% distilled water) was added to each well for removal the dye from lysosomes of live cells. Optical density_540_ of the washout with a spectrophotometer was measured that assessed live cell density whose percentage values were calculated.

### Comet assay

Single cell gel electrophoresis was carried out to study DNA damage of the cells treated with different concentrations of PbCl_2_ and lead acetate. Lymphocytes cultured with different concentrations of a Pb-chemical were harvested and used in the alkaline comet assay technique. Slides were coated with 1% agarose and were allowed for air drying. Pellets of lymphocytes, obtained by centrifugation of cultured cells were washed with PBS, and the pellet was mixed with three times the cell volume of the pellet with low melting point agarose (LMPA) 1% in sol state. The mixture of cells and LMPA sol was placed over the agarose coated slide that was kept at 4 °C for 10 min until the slide got dry. The dried slides were submerged into a pre-cooled lysing solution of the mixture of 0.4 M Tris HCl, 0.8 M dithiothreiotl (DTT) and 1% sodium dodecyl sulphate (SDS) of pH 7.5 and the mixture was kept at 4 °C in dark for about 30 min. The slides were subsequently removed and placed in another lysing solution with 0.4 M Tris HCl, 2 M NaCl, 1% SDS, 0.05 M EDTA at pH 7.5 for 30 min. The slides were placed in tris borate EDTA (TBE) buffer, which contained 89 mM Tris, 89 mM Boric acid and 2.5 mM EDTA at pH 8.3 for 10 min. The slides were transferred to a horizontal gel electrophoretic chamber with TBE buffer. Electrophoresis was carried out at 20 V and 12 mA for 12.5 min. After the electrophoresis, the slides were washed with 0.9% NaCl for 2.5 min, and the second electrophoresis was carried out at 20 V, 12 mA for 4 min in alkaline solution, which contained, 0.03 M NaOH and 1 M NaCl. The slides were placed in a neutralizing solution with 0.4 M Tris HCl for 5 min, and again the slides were washed in TBE buffer. After 5 min, the slides were stained with EB solution and were observed under the fluorescence microscope at 400×, for scoring the comets. Probits of observed lethality percentage values calculated from percent values of observed comets due to PbCl_2_ or lead acetate treatment; probits analyses of toxicity data were done.

## Results

Percent lethality (PL) values recorded from data sets of tests with TB, AO/EB, MTT and NRU assays were transformed to their probits were used to construct respective plots against Pb concentrations in log scale (Figure 3); plots were used for extrapolation to compute individual lethal concentration (LC) values, LC_25_, LC_50_ and LC_75_ in each method. The individual minimum inhibitory concentration (MIC), highest permissive concentration (HPC) and LC_100_ values were noted directly from experiments. Data from all the toxicity monitoring experiments were summarized for probit analysis of toxicity and all LC values were cited (Tables 1–4).

**Table 1:** Probit transformations of percent lethality values during PbCl_2_ toxicity to human lymphocytes growing in DME medium, assessed by four methods, TB, AO/EB staining, MTT assay, NRU assay and comet assay.

PbCl_2_(ppm)	Log_10_ concen-tration of PbCl_2_	PL of cells by TB staining	Probits of TB staining	PL of cells by AO/EB staining	Probits of AO/EB staining	PL of cells by MTT assay	Probits of MTT assay	PL of cells by NRU assay	Probits of NRU assay	DFI (%)	Probits of DFI
0	0	0	0	0	0	0	0	0	0	0	0
300	2.47	8.3	3.6	12	3.8	8.6	3.6	4.5	3.3	9	3.6
600	2.77	19.8	4.2	25	4.3	15.8	3.9	16.9	4.0	21	4.2
900	2.95	24.0	4.3	36	4.6	23.2	4.2	28.1	4.4	35	4.6
1200	3.07	36.6	4.6	42	4.7	41.3	4.7	40.5	4.7	41	4.7
1500	3.17	48.4	5.9	54	5.1	49.4	4.9	51.7	5.0	55	5.1
1800	3.25	60.3	5.2	70	5.5	59.5	5.2	64.1	5.3	69	5.4
2100	3.32	73.5	5.6	82	5.9	68.1	5.4	67.5	5.4	83	5.9
2400	3.38	85.0	6.0	89	6.2	86.3	6.1	79.8	5.8	91	6.3
2700	3.43	92.2	6.4	94	6.5	96.1	6.7	91.1	6.3	96	6.7
3000	3.47	100	-	100	-	98.6	7.1	93.3	6.4	100	-
3300	3.51	_-_	_-_	_-_	_-_	100	_-_	98.9	72	_-_	_-_

Note: TB, Trypan blue; AO/EB, Acridine orange/ethidium bromide; DMEM, Dulbecco's modified Eagle's medium; MTT, 3-[4, 5- dimethylthiazol-2-yl] 2,5-diphenyl tetrazolium bromide; NRU, Neutral red uptake assay; PL, Percent lethality; DFI, DNA fragmentation index; -, Not applicable. Experiments were repeated thrice and the last data set was presented.

**Table 2:** Toxicity values of PbCl_2_ to human lymphocytes obtained by experimentation and the probit computation.

	Toxicity values (mg/L)					
Assay methods	MIC*	LC_25_	LC_50_	LC_75_	HPC*	LC_100_*
TB staining	300	831.76	1318.2	1995.3	2700	3000
AO/EB staining	300	758.57	1174.9	1698.2	2700	3000
MTT assay	300	691.83	1348.9	2344.2	3000	3300
NRU assay	300	707.94	1258.9	1995.3	3000	3300
Comet assay	300	812.83	1258.9	1737.8	2700	3000

* Note: *From experiments

**Table 3:** Probit transformations of percent lethality values during CH_3_COOPb toxicity to human lymphocytes growing in DMEM, assessed by four methods, TB, AO/EB staining, MTT assay, NRU assay and comet assay.

Conc. of CH_3_COOPb (mg/L)	Log_10_ concentration of CH_3_COOPb	PL of cells by TB staining	Probits of TB staining	PL of cells by AO/EB staining	Probits of AO/EB staining	PL of cells by MTT assay	Probits of MTT assay	PL of cells by NRU assay	Probits of NRU assay	DFI (%)	Probits of DFI
0	-	0	-	0	-	0	-	-	-	-	-
150	2.17	16	4.0	12	3.8	13.6	3.9	12.1	3.8	11	3.7
300	2.47	24	4.3	21	4.2	20.2	4.2	20.3	4.2	20	4.1
450	2.65	39	4.7	33	4.6	34.6	4.6	32.3	4.5	32	4.5
600	2.77	47	4.9	46	4,9	39.1	4.7	40.5	4.7	47	4.9
750	2.87	56	5.2	52	5.1	59.5	5.2	51.9	5.0	53	5.1
900	2.95	67	5.4	61	5.3	77.1	5.7	62.3	5.3	64	5.3
1050	3.02	79	5.8	74	5.6	85.3	6.0	74.9	5.7	76	5.7
1200	3.07	86	6.1	89	6.2	94.4	6.6	84.7	6.0	88	6.1
1350	3.13	93	6.5	96	6.7	97.2	6.9	94.0	6.5	94	6.5
1500	3.17	100	-	100	-	98.3	7.1	97.9	7.0	100	-

Note: TB, Trypan blue; AO/EB, Acridine orange/ethidium bromide; DME medium, Dulbecco’s modified Eagle’s medium; MTT, 3-[4, 5- dimethylthiazol-2-yl] 2,5-diphenyl tetrazolium bromide; NRU, Neutral red uptake assay; PL, Percent lethality; DFI, DNA fragmentation index; –, Not applicable. Experiments were repeated thrice and the last data set was presented.

**Table 4:** Toxicity values of CH_3_COOPb to human lymphocytes obtained by experimentation and the probit computation.

	Toxicity values (mg/L)					
Assay methods	MIC*	LC_25_	LC_50_	LC_75_	HPC*	LC_100_*
TB staining	150	295.12	588.84	851.13	1350	1500
AO/EB staining	150	338.84	512.86	954.99	1350	1500
MTT assay	150	371.53	524.81	707.94	1350	1500
NRU assay	150	323.59	501.18	707.94	1350	1500
Comet assay	150	331.13	512.86	758.57	1350	1500

* Note: *From experiments

### TB staining

**Figure 5:**
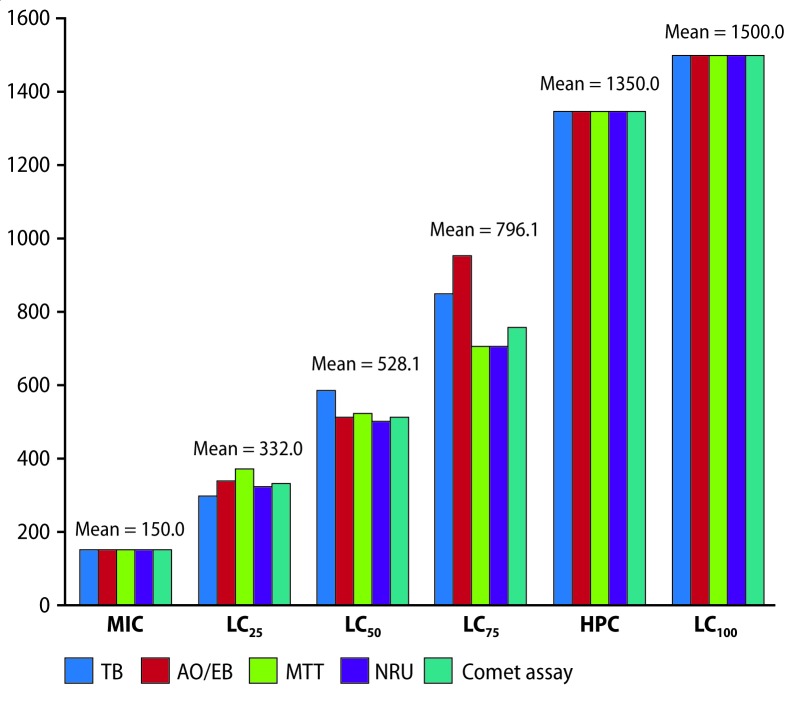
Histogram of MIC, LC_25_, LC_50_, LC_75_, HPC and LC_100_ values corresponding to TB, AO/EB, MTT, NRU and comet assay methods (see text for abbreviations) after the cells treated with lead acetate. M= mean value (mg/L).

TB staining indicated that cell counts decreased gradually after growing lymphocytes with PbCl_2_ at graded levels of 300 to 3000 mg/L, whereas similar results of decreased pattern of live cell count from 150 to 1500 mg/L level with lead acetate. The MIC value was recorded as 300 mg/L PbCl_2,_ HPC and LC_100_ values were 2700 and 3000 mg/L PbCl_2,_ respectively; whereas MIC, HPC and LC_100_ values were 150, 1350 and 1500 mg/L lead acetate, respectively. From the probit plot, it was ascertained that for values of LC_25,_ LC_50_ and LC_75_, for probit values, 4.33, 5.00 and 5.67, respectively, the corresponding log_10_ concentration values were 2.92, 3.12 and 3.30, respectively for PbCl_2_ (Figure 4, Table 2); whereas for lead acetate, these values were 2.47, 2.77 and 2.93, respectively. Antilog values of these log_10_ concentration values were the following LC values, 831.76 (LC_25_), 1318.25 (LC_50_), 1995.26 (LC_75_) mg/L PbCl_2_ and 295.12 (LC_25_), 588.84 (LC_50_), 851.13 (LC_75_) mg/L lead acetate (Figure 5, Table 4).

### AO/EB staining

**Figure 6:**
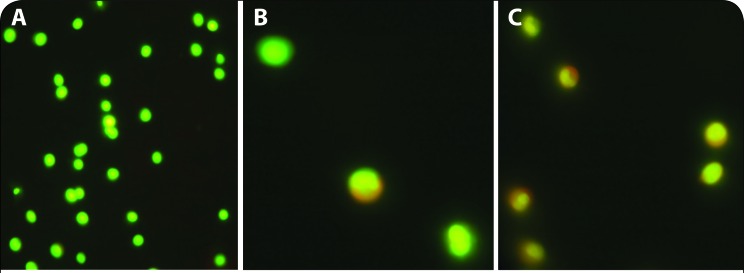
AO/EB staining. Control cells (A); Cells after treated with 1500 mg/L PbCl_2_(B); Cells after treated with 1500 mg/L lead acetate (C).

Treatment of cells with different concentrations of PbCl_2_ for 24 hr resulted in a decreasing pattern of living cell counts (Figure 6). The number of dead cells gradually increased from the level of 300 to 2700 mg/L level, and it was found that there were no cells at the 3000 mg/L level. In case of lead acetate, the decreasing pattern of live cell count started from 150 to 1350 mg/L level, whereas there were no cells at 1500 mg/L. Experimentally, the lethality was seen at 300 mg/L, which was recorded as the MIC value, the HPC was 2700 mg/L, and the LC_100_ value was 3000 mg/L for PbCl_2_; and for lead acetate the MIC, HPC and LC_100_ values were 150, 1350 and 1500 mg/L, respectively. From the plot, for PbCl_2_ it was ascertained that for values of LC_25_, LC_50_, and LC_75_, the corresponding log_10_ concentration values were 2.88, 3.07 and 3.23, respectively (Figure 4, Table 2); and for lead acetate the values were 2.53, 2.71, 2.98, respectively. Antilog values of these log_10_ concentration values are 758.57 (LC_25_), 1174.89 (LC_50_) and 1698.24 (LC_75_) mg/L, which were the computed LC values of PbCl_2_, and for lead acetate the computed LC values were 338.84 (LC_25_), 512.86 (LC_50_) and 954.99 (LC_75_) mg/L (Figure 5, Table 4).

### MTT assay

The cell density at OD_570_ gradually decreased from the level of 300 mg/L to 3000 mg/L and it was found that there were no cells at the 3300 mg/L PbCl_2_ level. Experimentally, the MIC value was 300 mg/L, whereas the HPC was 3000 mg/L, and the LC_100_ value was 3300 mg/L PbCl_2_. With lead acetate, the MIC was 150, whereas the HPC and LC_100_ values were 1350 and 1500 mg/L. Extrapolated log_10_ values of PbCl_2_ from the plot were 2.84, 3.13 and 3.37, respectively as LC_25,_ LC_50_, and LC_75_ log_10_ values (Figure 4, Table 2); whereas similar values for lead acetate were 2.57, 2.72, 2.85, respectively. Thus, these log_10_ concentration values generated LC values: 691.83 (LC_25_), 1348.96 (LC_50_) and 2344.22 mg/L (LC_75_) for PbCl_2,_ and 371.53 (LC_25_), 524.81 (LC_50_), 707.94 mg/L (LC_75_) for lead acetate (Figure 5, Table 4).

### NRU assay

Cell density, monitored at OD_540_ gradually decreased from the level of 300 to 3300 mg/L PbCl_2_. Experimentally, the MIC value was 300 mg/L; whereas the HPC was 3000 and the LC_100_ was 3300 mg/L PbCl_2_. Log_10_ values of PbCl_2_ concentrations extrapolated from the probit plot yielded log_10_ values 2.85, 3.10 and 3.30, for values of LC_25,_ LC_50_, and LC_75_, respectively (Figure 4, Table 2); and for lead acetate these values were 2.51, 2.70 and 2.85, respectively. Further, these log_10_ concentration values generated LC values: 707.94 (LC_25_), 1258.92 (LC_50_) and 1995.26 mg/L (LC_75_) PbCl_2_, and 323.59 (LC_25_), 501.18 (LC_50_), 707.94 mg/L (LC_75_) lead acetate (Figure 5, Table 4).

### Comet assay

**Figure 7:**
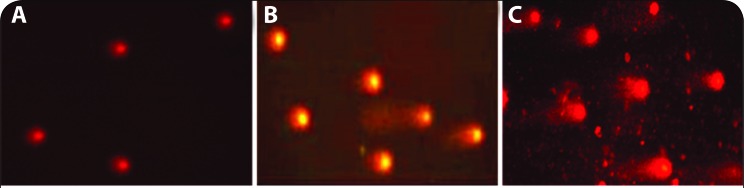
Comet assay. Control cells (A); Cells after treated with 1500 mg/L PbCl_2_ (B); Cells after treated with 1500 mg/L lead acetate (C).

It was observed that, comet tail was found increasing in cells treated with 300 mg/L to 3000 mg/L PbCl_2_ and 150 to 1500 mg/L for lead acetate (Figure 7); DNA fragmentation index (DFI) too increased with increasing gradations of both lead chemicals (Tables 1 and 3). From probit analysis, it was found that LC_25_, LC_50_ and LC_75_ values were 812.83, 1258.9 and 1737.8 mg/L PbCl_2_, respectively (Figure 4, Table 2); whereas similar values were 331.13, 512.86, 758.57, respectively for lead acetate. The MIC value as 300, the HPC value as 2700 and the LC_100_ value as 3000 mg/L PbCl_2_, were recorded experimentally while, similar values were 150 (MIC) and 1350 (HPC), 1500 (LC_100_) mg/L for lead acetate (Figure 5, Table 4).

## Discussion

This work demonstrated that four methods of monitoring cytotoxicity of lymphocytes, namely TB staining, AO/EB staining, MTT assay and NRU assay could give dependable results. The MIC value in each method were invariably 300 mg/L; the LC_25_ values were almost in an agreeable range: 691.83 to 831.76 mg/L; LC_50_ values were in the range: 1174.9 to 1348.9 mg/L; LC_75_ values were in the range: 1698.2 to 2344.2 mg/L; HPC values were in the range: 2700 to 3000 mg/L; LC_100_ values were in the range: 3000 to 3300 mg/L, including the results obtained for comet assay attempts, for PbCl2. Similarly, The MIC value in each method were invariably 150 mg/L; the LC_25_ values were almost in an agreeable range: 295.12 to 371.53 mg/L; LC_50_ values were in the range: 501.18 to 588.84 mg/L; LC_75_ values were in the range: 707.94 to 954.99 mg/L HPC values was 1350 mg/L in all assays; LC_100_ values was 1500 mg/L in all assays, including the results obtained for comet assay attempts, for lead acetate. It was recorded that each assay method monitored for cytotoxicity were in same range monitored for a chemical; but, separate ranges for each chemical were noted. Cytotoxicity and genotoxicity values due to each chemical were in the same range for a chemical.

Many toxicology test systems are undependable in animal systems as the exemplary mouse or rabbit or guinea pig systems are not true representative of a human body. Therefore, there are large gaps in the mirror of toxicity models with animals (Tzimas 1994); nonetheless, all popular animal models are mammals. Further, the requirement of a high number of animals in toxicology are so much for chemicals like 30,000 or more, which discourages the use of animals in assay systems (Gilbert 2010). Therefore the use of pluripotent stem cells (PSCs) and related cell lines in toxicology had been well-recognized (Rosler *et al*., 2004). It is consensus that, the animal system has a holistic approach in toxicity studies that is unavailable in cell cultures — a fact that supports the use of whole animal models.

However, animals tests can never be adequately standardized like human cellular systems, as the experiment should have growing cells under controlled conditions – *in vitro* human cell lines. In fact, the use of PSCs and their derivatives in toxicology has been well recognized (reviewed by Laustriat *et al*., 2010). PSCs have unique capacity of self-renewal in differentiation with advantages over somatic cells and those could be grown *in vitro,* as permanent cell lines. Moreover, surplus embryos from an *in vitro* fertilization unit could lend to the initiation of individual cell lines (Loser *et al*., 2010), during toxicity testing. Reprogramming murine fibroblasts by viral transfer of 4 genes, associated with pluripotency could be induced, thus PSCs could be created (Takahashi & Yamanaka, 2006). These workers could demonstrate that these specialized somatic cells could be reversed in PSCs *in vitro*.

The use of induced pluripotent stem cells (iPSCs) is a reliable technology for the development of different cell lines. The use of iPSC along with bio-monitoring data could enable a proper understanding of environmental metal/chemical toxicity. Thus, the iPSC technology has a tremendous potential for the development of predictive toxicology. Developmental toxicity has slowly become an important area of future assessment (Laustriat *et al*., 2010). Indeed, we are unaware of rising frequencies of many ailments, for example, cancer, autism, changes in pubertal timings are the areas not clearly known, with problem from environmental pollution, for example. In fact, if the toxicity pathways that induce DNA damage and affect DNA repair and other associated genetic process, predictive toxicology would bring new ideas in cancer mechanism due to chemicals of environmental concerns that could aid in the identification and classification of carcinogens (Grimsrud & Andersen, 2010). By the by, toxic effect on immune pathways could be elucidated (Guzik *et al*., 2001). Genotoxicity testing of 131-radioiodine a drug used for treatment of patients with thyroid diseases with cultured human lymphocytes was recently described (Hosseinimehr *et al*., 2013), as a work in predictive toxicology.

It has been consensus that hematopoietic /progenitor cells are more in UCB compared to bone marrow blood, as the later is involved in adults. Thus, this toxicity work using cord blood lymphocytes overrides the use of bone marrow stem cells in studies with predictive toxicology. In a study with human UCB, it was estimated that contents of nucleolar cells, granulocyte macrophage, erythroid and multipotent progenitor cells are mainly viable for at least three days under 4 °C or 21 °C (Broxmeyer *et al*., 1989). This study involves storage of the cells at 4 °C and further work at 22 °C. Thus, essential constituents of cord blood relating to stem cell would not be destroyed during the period of the study. Moreover, microarray technology involving environmental toxicants for understanding the underlying toxicity has been defined; to identify toxicant with specific genetic markers, such studies with heavy metals have been initiated (Bartosiewicz *et al*., 2001). Indeed, metals cause oxidative stress in cells, which is well studied in microarray system.

## Conclusion

Lead acetate with the LC_100_ value around 1500 mg/L was more toxic than lead chloride with the LC_100_ value around 3300 mg/L to human lymphocytes. LC_50_ values obtained by probit analyses were accordingly different for each chemical. Cyto-toxicity and Geno-toxicity values due to each chemical were in the same range for a chemical.
